# Ciclopirox Inhibition of eIF5A Hypusination Attenuates Fibroblast Activation and Cardiac Fibrosis

**DOI:** 10.3390/jcdd10020052

**Published:** 2023-01-29

**Authors:** Kadiam C. Venkata Subbaiah, Jiangbin Wu, Wai Hong Wilson Tang, Peng Yao

**Affiliations:** 1 Aab Cardiovascular Research Institute, Department of Medicine, University of Rochester School of Medicine & Dentistry, Rochester, NY 14642, USA; 2 Department of Cardiovascular Medicine, Cleveland Clinic, Cleveland, OH 44195, USA; 3 Department of Biochemistry & Biophysics, University of Rochester School of Medicine & Dentistry, Rochester, NY 14642, USA; 4 The Center for RNA Biology, University of Rochester School of Medicine & Dentistry, Rochester, NY 14642, USA; 5 The Center for Biomedical Informatics, University of Rochester School of Medicine & Dentistry, Rochester, NY 14642, USA

**Keywords:** collagen, ciclopirox, ECM, eIF5A, fibroblast, fibrosis, heart failure, translational control

## Abstract

Cardiac fibrosis is a primary contributor to heart failure (HF), and is considered to be a targetable process for HF therapy. Cardiac fibroblast (CF) activation accompanied by excessive extracellular matrix (ECM) production is central to the initiation and maintenance of fibrotic scarring in cardiac fibrosis. However, therapeutic compounds targeting CF activation remain limited in treating cardiac fibrosis. Eukaryotic translation initiation factor 5A (eIF5A), upon being hypusinated, is essential for the translation elongation of proline-codon rich mRNAs. In this study, we found that increased hypusinated eIF5A protein levels were associated with cardiac fibrosis and heart dysfunction in myocardial infarction (MI) mouse models. Ciclopirox (CPX), an FDA-approved antifungal drug, inhibits the deoxyhypusine hydroxylase (DOHH) enzyme required for eIF5A hypusination. Results from preventive and reversal mouse models suggest that CPX treatment significantly reduced MI-driven cardiac fibrosis and improved cardiac function. In vitro studies of isolated mouse primary CFs revealed that inhibition of eIF5A hypusination using CPX significantly abolished TGFβ induced CF proliferation, activation, and collagen expression. Proteomic analysis from mouse CFs reveals that CPX downregulates the expression of proline-rich proteins that are enriched in extracellular matrix and cell adhesion pathways. Our findings are relevant to human heart disease, as increased hypusinated eIF5A levels were observed in heart samples of ischemic heart failure patients compared to healthy subjects. Together, these results suggest that CPX can be repurposed to treat cardiac fibrosis and ischemic heart failure.

## 1. Introduction

Cardiac fibrosis is a critical event during heart failure and cardiac pathological remodeling [1]. Pro-fibrotic protein synthesis is a hallmark of cardiac fibrosis, and translational activation of the synthesis of these proteins has been reported to contribute to the progression of heart disease [2]. Translational activation of synthesis of proline-rich (PRR) pro-fibrotic protein synthesis by glutamyl-prolyl-tRNA synthetase (EPRS) has been considered a critical mechanism of enhancing cardiac fibroblast (CF)-to-myofibroblast (MF) transdifferentiation [3]. Halofuginone, a prolyl-tRNA synthetase-specific inhibitor, significantly inhibits PRR protein expression and reduces cardiac fibrosis in multiple mouse heart failure models, such as ischemia/reperfusion [4]. More translation factor inhibitors need to be tested to provide proof-of-principle evidence to support the concept of using translation inhibitors to treat cardiac fibrosis and heart disease.

The human translation machinery is comprised of three major parts: aminoacyl-transfer RNAs (tRNAs), ribosomes, and translation factors (initiation, elongation, and termination factors) [5]. EPRS provides ribosomes with prolyl-tRNA^Pro^ substrates for proline genetic codon decoding and PRR mRNA translation. Besides EPRS, eIF5A (eukaryotic initiation factor 5A) is indeed a translation elongation factor in eukaryotes required for efficient peptide bond formation for Pro dipeptidyl motifs for PRR proteins [6,7,8,9]. EF-P (Elongation factor P) is a homolog of eIF5A in prokaryotes. eIF5A/EF-P is evolutionarily conserved across three major life kingdoms in Bacteria, Archaea, and Eukarya. Hypusination is a process of converting deoxyhypusine to hypusine [10]. eIF5A hypusination on a Lys^50^ residue is essential for its catalytic function. eIF5A Lys^50^ hypusination forms during two-step enzymatic reactions mediated by deoxyhypusine synthase and deoxyhypusine hydroxylase (DOHH). DOHH is an iron-dependent enzyme that catalyzes the conversion of deoxyhypusine to hypusine for eIF5A maturation. eIF5A plays a vital role in inflammation, such as macrophage activation [11], B cell immunity [12], and cancer progression [13]. Ciclopirox (CPX), a deoxyhypusine hydroxylase inhibitor, is an FDA-approved drug to treat fungus infections [14]. CPX has been recently reported to have promising potential to treat other human diseases, including diabetes [15], cardiac disorders [16], inflammation [14], cancer [17], and HIV infection [18]. However, the potential effects of CPX in regulating cardiac fibrosis and protein expression in CFs remain unclear.

Here we examined the anti-fibrotic effects of CPX in a mouse heart failure model of myocardial infarction (MI). We show that eIF5A hypusination is induced in human and mouse failing hearts upon MI stress. CPX treatment in MI mice reduces cardiac hypertrophy, antagonizes fibrosis, and restores cardiac functions in both preventive and reversal models. Inhibition of eIF5A hypusination by CPX reduces extracellular matrix protein expression in mouse primary CFs. Finally, we show that CPX inhibits proliferation, migration, and CF-to-MF activation using primary mouse CF cultures. Mechanistically, CPX inhibition of expression of Pro-rich proteins such as collagens and ATF4 contributes to the cellular phenotypic changes in CFs.

## 2. Methods

### 2.1. Human Specimens

All human samples of frozen cardiac tissues, including 10 samples from explanted ischemic failing hearts and 10 samples from donor non-failing hearts, and paraffin-embedded section slides from ischemic heart failure (ISHF), or no-failing donor (NF) hearts, were acquired from the Cleveland Clinic. The Cleveland Clinic Institutional Review Board approved the study protocol and all participants provided written informed consent. According to the rules of the Declaration of Helsinki, this study was approved by the Material Transfer Agreement between the University of Rochester Medical Center (URMC) and the Cleveland Clinic.

### 2.2. Mice

The C57BL/6J wild type (WT) mice were purchased from the Jackson Laboratory. For animal experiments, WT mice, with the same age and gender, from littermates or sibling-mating were used. All animal procedures were performed following the National Institutes of Health (NIH) and the University of Rochester Institutional Guidelines. The animal studies were approved by the University of Rochester University Committee on Animal Resources (Protocol # 102279/2016-013E). For heart disease models, the left anterior descending (LAD) coronary artery ligation derived myocardial infarction model of HF was used.

### 2.3. Reagents, Antibodies, Plasmids, and siRNAs

The type II collagenase was purchased from Worthington company (Cat. No. LS004177) (Lakewood, NJ, USA). Taurine (Cat. No. 1665100) was purchased from Acros Organics (Geel, Belgium). The antifade mounting medium with DAPI (Cat. No. H-1500) was obtained from Vectorlabs (Newark, CA, USA). Antibodies used in this study (Table 1):

### 2.4. Mouse Heart Failure Models

The University of Rochester Medical Center Animal Care and Use of Committee approved all experimental animal procedures (Protocol # 102279/2016-013E). Mice were procured at 8 weeks of age and maintained in a vivarium facility with ad libitum free access to standard chow and water. In this study, we used myocardial infarction (MI) surgery (left anterior descending coronary artery ligation). For the entire study, we used age-matched male mice at ~8–12 weeks. All the mouse surgeries were performed by the mouse Microsurgical Core facility at URMC. We used 6–8 mice for individual treatment groups to get the statistical significance of the different groups in this study.

The LAD ligation-based MI surgery was performed by the Mouse Microsurgical Core of Aab CVRI [3]. For MI surgery, male or female mice were placed on a heating pad, and the airway was stabilized by endotracheal intubation and mechanical ventilation provided (inspiratory tidal volume of 250 μL at 130 breaths/min). The mice were given SR buprenorphine 2.5 mg/Kg via subcutaneous injection. Isoflurane flow was continually maintained at approximately 1.5%, along with oxygen. A midline cervical incision was made to expose the trachea for intubation with a PE90 plastic catheter. The catheter was connected to a Harvard mini vent supplying supplemental oxygen with a tide volume of 225–250 μL and a respiratory rate of 130 strokes/min. Surgical plane anesthesia was subsequently maintained at 1–1.5% isoflurane. The skin was incised, and the chest cavity opened at the level of the 4th intercostal space. Oral intubation was employed by placing PE 90 tubing in the mouth and advancing slowly into the trachea. Mechanical P.I. ventilation (tidal volume of approximately 0.4 mL at 130 breaths/min) was then begun. After intubation, a midline incision was made between the sternum and the left internal mammalian artery. Alternatively, a lateral incision (left thoracotomy) was made in the fourth intercostal space. The mouse heart was exposed, and the left coronary artery branch points were visualized under 10× magnification before ligation. The LAD coronary artery was ligated intramurally 2 mm from its ostial origin for standard MI with a 9-0 proline suture. Transmural ischemia was assured by color loss on the left ventricle wall and ST-segment elevation, which was noted on the electrocardiogram. The lungs were inflated and the chest was closed in two layers; the ribs (inner layer) were closed with 6-0 coated vinyl sutures in an interrupted pattern. The skin was closed using 6-0 nylon or silk sutures in a subcuticular manner. The anesthesia was stopped, and once the mouse was breathing on its own, the mouse was removed from the ventilator and allowed to recover in a clean cage on a heated pad. A sham operation was performed using the same procedure, but a suture was passed under the LAD coronary artery without ligation.

For mouse experiments, age/sex/genetic background matched mice were randomly separated into indicated groups. MI surgery and echocardiography measurement were performed blindly by the Microsurgical Core surgeons. The Histology Core prepared the heart sections. For group size justification, we have achieved a power analysis using G*power version 3.1.9.6. The assumptions include the same standard variance in each study group, effect size=$\frac{\mathrm{Difference}\;\mathrm{of}\;\mathrm{the}\;\mathrm{means}\;\mathrm{between}\;\mathrm{study}\;\mathrm{groups}}{\mathrm{common}\;\mathrm{standard}\;\mathrm{deviation}}$, alpha level = 0.05, power = 0.9, and the number of study groups. The effect size for specific experiments is assumed based on similar studies and literature. As an exemplary experiment, the standard deviation for weight after MI treatment is about 10%. The minimum difference to be considered significant is 25% in MI-induced cardiac hypertrophy and heart weight gain. With an overall type I error rate (alpha level) of 5%, at least 5 mice per treatment group are required to achieve 90% power to detect the difference in heart weight. In previous experiences from our Microsurgical Core, we have observed a survival rate of ~90% after the MI procedure. To offset the possible loss of one mouse per treatment, we used at least 6 mice per treatment group. In rare cases, mice might die after surgery, which reduces the number of mice.

### 2.5. Preventive and Reversal Model Using CPX

We performed an intraperitoneal (i.p.) injection of CPX at 2.5 mg/Kg/day for 14 days, one day and six days post-MI surgery in a preventive model and a reversal model, respectively. Ciclopirox was prepared in combination of saline, Polyethylene glycol (PEG) 400 and ethanol (60:35:5 in volume) for the animal injections. We have conducted pilot tests on CPX dosage determination in animal models ranging from 1.25, 2.5, 5, and 10 mg/Kg body weight [19]. All these four CPX concentrations were tested in WT mice with MI surgery for 2 weeks. We found that 2.5 mg/Kg body weight of CPX significantly reduced collagen deposition and cardiomyocyte hypertrophy, when compared to the saline treated control group, indicating the highest benefit versus side effect ratio.

### 2.6. Echocardiography

For the sham and MI surgical mouse models, B-mode long-axis echocardiographic image collection was performed using a Vevo2100 echocardiography machine (VisualSonics, Toronto, ON, Canada) and a linear-array 40 MHz transducer (MS-550D, Fujifilm, Minato City, Tokyo). Heart rate was monitored during echocardiography measurement. Image capture was performed in mice under general isoflurane anesthesia with a heart rate maintained at around 550–650 beats/min. The HR could vary in individual mice due to the potential effect of anesthesia or the surgeon’s operation variation. LV systolic and diastolic measurements were captured in B-mode from the parasternal long axis. Fraction shortening (FS) was assessed as follows: %FS = (end diastolic diameter—end systolic diameter)/(end diastolic diameter) × 100%. Left ventricular ejection fraction (EF) was measured and averaged in both the parasternal short axis (M-Mode) using the tracing of the end diastolic dimension (EDD), and end systolic dimension (ESD) in the parasternal long axis: %EF = (EDD-ESD)/EDD. Hearts were harvested at multiple endpoints depending on the study. In addition to EF and FS, end systolic volume (LVESV) and end diastolic volume (LVEDV) were measured.

### 2.7. Adult Cardiac Fibroblasts Isolation and Culturing

The Langendorff perfusion system was used to isolate cardiac fibroblasts from the murine heart. Mice were fully anesthetized via intraperitoneal injection of ketamine/xylazine. Once losing pedal reflex, the mouse was secured in a supine position. The heart was excised, and blood was removed using perfusion buffer. The heart was then fastened onto the CM perfusion apparatus, where perfusion was initiated using the Langendorff mode. Our Langendorff perfusion and digestion consisted of three steps at 37 °C: 4 min with perfusion buffer (0.6 mM KH_2_PO_4_, 0.6 mM Na_2_HPO_4_, 10 mM HEPES, 14.7 mM KCl, 1.2 mM MgSO_4_, 120.3 mM NaCl, 4.6 mM NaHCO_3_, 30 mM taurine, 5.5 mM glucose, and 10 mM 2,3-butanedione monoxime), then 3 min with digestion buffer (300 U/mL collagenase II [Worthington] in perfusion buffer), and finally, perfusion with digestion buffer supplemented with 40 μM CaCl_2_ for 8 min. After perfusion, the ventricle was placed in a sterile 35 mm dish with 2.5 mL digestion buffer and shredded into several pieces with forceps. A 5 mL stopping buffer (10% FBS, 12.5 μM CaCl_2_ in perfusion buffer) was added and pipetted several times until tissues dispersed readily, and the solution turned cloudy. The cell solution was passed through a 100 μm strainer. Cardiac fibroblasts (CFs) from the supernatant were pelleted for 5 min at 1200 rpm at 4 °C. CFs were plated in 4–5 mL CF media (DMEM with 10% FBS and 1% penicillin/streptomycin) in a 60 mm plate, and were washed vigorously 3–5 times with 2 mL 1× PBS several times after 2–3 h to remove the unattached cells and debris. Then, they were replaced with fresh CF media. Alternatively, for CF-only isolation, pre-weaned mice were fully anesthetized. The heart was directly cut into small pieces and digested in the digestion buffer for 4 × 10 min at 37 °C with slow stirring, and CFs were plated the same as the Langendorff isolation of CFs.

### 2.8. Cell Culture and Transfection

NIH/3T3 cells were cultured in DMEM supplemented with 10% bovine calf serum (VWR) and 1% penicillin/streptomycin (ThermoFisher, Waltham, MA, USA). Primary CFs isolated from mouse hearts of both genders were cultured in DMEM supplemented with 10% FBS (ThermoFisher) and 1% penicillin/streptomycin. Primary cells were used at P0 for CF activation assays. We used the Polyjet to transfer the NIH/3T3 cells and DNA plasmids. siRNA transfection (100 nM) in primary CFs was performed using lipofectamine 3000, following the manufacturer’s instructions.

### 2.9. RNA Isolation and RT-qPCR

For heart tissues (human and mouse) or cell samples, the RNA extraction was performed using TRIzol reagent (ThermoFisher), following instructions in the manual, and was used to detect the expression of specific genes. Briefly, the tissues were homogenized in TRIzol using Minilys Personal Homogenizer (Bertin Technologies, Montigny-le-Bretonneux, France) and placed on ice for 15 min to lyse the tissue. Genomic DNA was removed using DNase I treatment followed by the phenol–chloroform–isoamyl alcohol extraction method. For the mRNA detection, 1 μg of total RNA was used as a template for reverse transcription using the iScript cDNA Synthesis Kit (Bio-Rad, Hercules, CA, USA). RT-qPCR was performed with cDNA, with primers of specific targets of interest, and with IQ SYBR Green Supermix (Bio-Rad). Data were analyzed using the formula of the ΔΔC(t) method. cDNA was used for detecting the expression of *Eif5a* and the marker genes, *Myh6*, *Myh7*, *Col1a1*, *Col3a1*, and *Fn1*. 18S rRNA or *Gapdh* was used as a normalization control for mRNA expression. The SYBR Green primer sequences or the Taqman probes are listed below.

SYBR green qPCR procedure: (1) Initial denaturation at 95 °C for 60 s. (2) 40 cycles of denaturation at 95 °C for 10 s and annealing/extension at 60 °C for 45 s. (3) Melt curve analysis by 0.5 °C increments at 5 s/step between 65 and 95 °C. qPCR primers used in this study (SYBR green) (Table 2).

### 2.10. Immunofluorescence Staining of Heart Tissue Sections and CFs

Mice were sacrificed, hearts were immediately removed, washed in ice-cold PBS, fixed in 10% neutral buffered formalin, and processed for paraffin-embedded sections in the Histological Core of Aab CVRI. Tissue sections were cut at a cross-section of 5 μm thickness, and 250 μm intervals were used for immunohistochemical analysis and to quantify the scar area. For immunofluorescence, paraffin-embedded slides were deparaffinized in a series of xylenes followed by 3 min of incubations in 100% ethanol and 95% ethanol. Then, they were placed in distilled water. Antigen retrieval was performed in citric acid buffer (pH 6.0), followed by quenching in 3% H_2_O_2_ in PBS for 30 min at RT. Sections were blocked in blocking buffer (2% BSA, 0.5% Triton X-100, 5% goat serum) for 2 h. Then, slides were incubated with primary antibodies (as listed in the antibody table) overnight at 4 °C. After primary antibody incubation, slides were washed in 1× PBS, followed by the secondary antibody (AlexFluor-488 or AlexFluor-594 conjugated) incubation in a blocking solution for 1 hr at room temperature (RT). Slides were then washed with 1× PBS (3 × 5 min) and mounted with DAPI (Vectorlabs, Newark, CA, USA), covered by coverslips, and air-dried (or kept in a PBS buffer inside before imaging). The images were obtained using the Olympus FV1000 (Olympus, Hong Kong, China) confocal microscope, and the intensity was measured by NIH Image J software. Four sections for each condition were used. For each sections 5–7, randomized fields of images were captured (4 sections × 7).

### 2.11. Picrosirus Red Staining

Paraffin-embedded heart tissue sections were deparaffinized, and the sections were incubated with Picrosirius red reagent (Abcam, Boston, MA, USA) for 1 h at RT. Slides were then washed with 1% acetic acid, followed by 100% ethyl alcohol, and mounted with a mounting medium. Images were captured using the Prime Histo XE Slide Scanner (Burlington, N.C., USA), and the fibrosis area was measured by Image J software (NIH, USA).

### 2.12. Wheat Germ Agglutinin (WGA) Staining

WGA staining was used to quantify the size of CMs in the murine heart. Deparaffinization, antigen retrieval, and quenching of auto-fluorescence were performed as described above. Heart tissue sections of mice from different treatments were probed with 10 μg/mL WGA-Alexa Fluor-488 (ThermoFisher) to stain the cardiomyocyte membrane for 1 hr at RT and followed by 3 × 5 min washes with 1× PBS. The slides were covered by coverslips with antifade solution (containing DAPI) for imaging. Cardiomyocytes were measured in the whole heart of vehicle- and CPX-treated mice and in remote and border zone areas of the hearts of MI mice. The images were taken in the fluorescence microscope, and the cross-sectional regions were quantified and measured using Image J Version 2.0. software (NIH, USA), using the hand drawing tool to outline the myocytes. Myocyte size was taken from images of at least 3–4 fields per heart; in total, 300–400 cells were measured.

### 2.13. Cardiac Fibroblast Activation Assay

Adult cardiac fibroblasts were isolated from ~2–3 months old WT mice and placed in 35 mm glass-bottom dishes. After 2 h, attached cells were washed with 1× PBS 3 times, changed to fresh CF culturing medium (DMEM containing 10% FBS and 1% penicillin/streptomycin), and cultured at 37 °C for 12 h (or overnight). Then, cells were treated with serum starvation for 12 h and stimulated with TGFβ (10 nM) for 24 h. Immediately, cells were fixed with 4% paraformaldehyde (PFA) for immunofluorescence staining, or lysed in TRIzol reagent for RNA isolation to detect the gene expression levels of myofibroblast activation markers by RT-qPCR. Cardiac fibroblast cells were isolated from WT mouse hearts and cultured for 24 or 48 h.

### 2.14. Cardiac Fibroblasts Migration Assay

To determine the cardiac fibroblast migration ability, 5 × 10^4^ isolated primary adult cardiac fibroblasts (ACFs) from WT mice were plated per well in 24-well plates. Fibroblasts were stimulated with human angiotensin II (100 nM) or were unstimulated (vehicle). Fibroblast monolayers were then scratched with a 200 μl pipet tip. To analyze the migration of the fibroblasts, the same scratched area was captured with the Olympus microscope after 0, 6, 12, and 24 h. The migration rate was calculated as cell-free area at 0 h—cell-free area at 6, 12, or 24 h)/cell-free area at 0 hr. The cell-free area was calculated using the Image J software (NIH, USA). The migration assay was carried out in 3 biological replicates with 3 wells per treatment condition.

### 2.15. Triphenyl Tetrazolium Chloride (TTC) Staining for Measurement of Infarct Size

Excised hearts were perfused with 1× PBS to remove the blood and sectioned into 4–5 levels (2 mm thick). The sliced hearts were placed in a petri dish with 1% TTC in 1× PBS and incubated for 15 min at 37 °C. Then, tissue slices were fixed with 10% formalin for 1 h. Hearts were visualized using a bright field microscope. Quantification of infarct size was performed using Image J Version 2.0. software (NIH, USA) by normalizing the total scar (white color area) to the left ventricle wall (% LV free wall) and averaging across four cross-sectional levels of the heart (apex to ligature).

### 2.16. Western Blot Analysis

Heart tissues and cultured CFs were homogenized in ice-cold RIPA lysis buffer with protease inhibitor cocktails (Santa Cruz). Cell debris was removed by centrifugation for 10 min at 10,000 rpm, 4 °C. Total protein concentration was determined by Bradford assay (Bio-Rad). An equal amount of protein was loaded onto 10% and 12% SDS-PAGE gels and then transferred to PVDF membranes. The membranes were blocked in the 5% milk in PBST buffer for 1 h at RT. The respective membranes were probed with specific primary antibodies for target proteins in 4% BSA (Sigma Aldrich, St. Louis, Missouri, United States) in PBST buffer overnight. After several washes with the PBST buffer, the blots were incubated with a horseradish peroxidase-conjugated secondary antibody in 3% milk with PBST buffer for 1 h and developed using the ECL reagent (Bio-Rad).

### 2.17. Quantitative Mass Spectrometry for Measuring Proteomic Changes

#### 2.17.1. Sample Preparation

For mass spectrometry experiments, vehicle and CPX-treated (10 mM for 24 h) primary mouse cardiac fibroblast cell lysate samples were run into a 4–12% SDS-PAGE gel for a short time to remove contaminants and create a ~10 mm length region, allowing the total protein to be evaluated in a single gel digest. After staining with SimplyBlue SafeStain (Invitrogen), these regions were excised, cut into 1 mm cubes, de-stained, and reduced and alkylated with DTT and IAA, respectively (Sigma). Gel pieces were dehydrated with acetonitrile. Aliquots of trypsin (Promega, Madison, Wisconsin, United States) were reconstituted to 10 ng/μL in 50 mM ammonium bicarbonate and added so that the solution was just covering the dehydrated gel pieces. After 0.5 h at room temperature (RT), additional ammonium bicarbonate was added until the gel pieces were completely submerged and placed at 37 °C overnight. Peptides were extracted the next day by adding 0.1% TFA, and 50% acetonitrile and dried down in a CentriVap concentrator (Labconco, Kansas City, MO, USA). Peptides were desalted with homemade C18 spin columns, dried again, and reconstituted in 0.1% TFA.

#### 2.17.2. LC-MS/MS

Peptides were injected onto a homemade 30 cm C18 column with 1.8 μm beads (Sepax, Newark, CA, USA), with an Easy nLC-1000 HPLC (ThermoFisher Scientific), connected to a Q Exactive Plus mass spectrometer (ThermoFisher Scientific). Solvent A was 0.1% formic acid in water, while solvent B was 0.1% formic acid in acetonitrile. Ions were introduced to the mass spectrometer using a Nanospray Flex source operating at 2 kV. The gradient began at 3% B and held for 2 min, increased to 30% B over 41 min, increased to 70% over 3 min and held for 4 min, then returned to 3% B in 2 min and re-equilibrated for 8 min, for a total run time of 60 min. The Q Exactive Plus was operated in a data-dependent mode, with a full MS1 scan followed by 10 data-dependent MS2 scans. The full scan was performed over a range of 400–1400 *m*/*z*, with a resolution of 70,000 at *m*/*z* of 200, an AGC target of 1 × 10^6^, and a maximum injection time of 50 ms. Ions with a charge state between 2 and 5 were picked for fragmentation. The MS2 scans were performed at 17,500 resolution, with an AGC target of 5e4 and a maximum injection time of 120 ms. The isolation width was 1.5 *m*/*z*, with an offset of 0.3 *m*/*z*, and a normalized collision energy of 27. After fragmentation, ions were put on an exclusion list for 15 s to allow the mass spectrometer to fragment lower abundant peptides.

#### 2.17.3. Data Analysis

Raw data from MS experiments were searched using the SEQUEST search engine within the Proteome Discoverer software platform, version 2.2 (ThermoFisher Scientific, Waltham, MA, USA), and the SwissProt human database. Trypsin was selected as the enzyme allowing up to 2 missed cleavages, with an MS1 mass tolerance of 10 ppm. Samples run on the Q Exactive Plus used an MS2 mass tolerance of 25 mmu. Carbamidomethyl was set as a fixed modification, while methionine oxidation was set as a variable modification. The Minora node was used to determine relative protein abundance between samples using the default settings. The percolator was used as the FDR calculator, filtering out peptides with a q-value greater than 0.01.

### 2.18. Statistical Analysis

All quantitative data were presented as mean ± SEM and analyzed using Prism 7 software (GraphPad). For a comparison between 2 groups, data were checked for normal distribution using the Kolmogorov–Smirnov test, and a Student *t* test was performed. For multiple comparisons among ≥3 groups with two variables, 2-way ANOVA with Tukey’s multiple comparisons test was performed. Statistical significance was assumed at a value of *p* < 0.05.

## 3. Results

### 3.1. eIF5A Protein Expression and Modification in Human and Mouse Heart Failure

**Figure 1:**
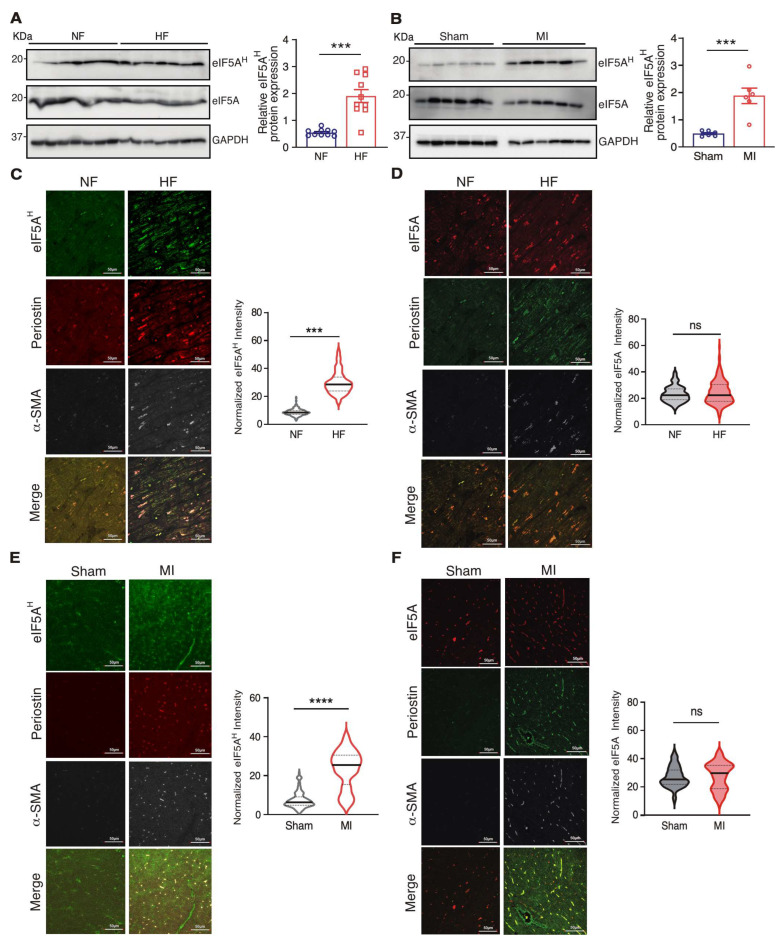
Induced eIF5A^H^ protein expression is associated with human and mouse heart failure. (**A**) eIF5A^H^ protein was increased in failing human hearts (*n* = 10) compared to non-failing donor hearts (*n* = 10). GAPDH was used as a loading control for quantification. (**B**) eIF5A^H^ protein was increased in mouse MI hearts (*n* = 6) compared to sham hearts (*n* = 6). Quantification of Western blot results from the border zone of the MI heart and the corresponding region of the sham heart was performed. GAPDH was used as a loading control for quantification. (**C**,**D**) eIF5A^H^ protein was increased in the cardiac fibroblasts of failing human hearts compared to non-failing donor hearts. *n* = 120 cells were counted from multiple sections from mouse hearts (*n* = 10). Scale bar: 10 μM. Co-staining of Postn and α-SMA was performed. (**E**,**F**) eIF5A^H^ protein was increased in the cardiac fibroblasts of mouse MI hearts (*n* = 6) compared to sham hearts (*n* = 6). Scale bar: 10 μM. *** *p* < 0.001, **** *p* < 0.0001, ns: not significant by unpaired two-tailed student *t* test.

eIF5A is not transcriptionally upregulated in TGFβ-activated human CFs from heart disease patients [2] or end-stage failing human hearts [20]. We measured the protein expression of eIF5A and post-translational modification of hypusinated eIF5A in non-failure donor hearts and failing hearts by Western blotting in total heart lysates. We observed an increase in the hypusinated form of eIF5A (eIF5A^H^) by ~3.5 folds in failing hearts compared to non-failing hearts (Figure 1A, Table 1). To examine whether similar changes occur in the early stage of heart disease, we performed left anterior descending coronary artery ligation to produce myocardial infarction (MI) in wild-type (WT) mice. We measured eIF5A protein expression in the heart tissues harvested 14 days post-surgery. We found a consistent increase in the hypusinated form of eIF5A by ~5 folds in the mouse MI hearts at the border zones (Figure 1B, Table 1). Interestingly, the modified form eIF5A^H^ was more highly expressed in activated myofibroblasts, as indicated by increased eIF5A^H^ (not eIF5A total protein) in Postn and α-SMA positive cells in both failing human (Figure 1C,D) and mouse (Figure 1E,F) heart tissues. These data indicate that activated eIF5A^H^ is induced in cardiac pathological remodeling and may contribute to cardiac disease occurrence.

### 3.2. Ciclopirox Inhibition of eIF5A Prevents Pathological Remodeling and Cardiac Fibrosis

**Figure 2:**
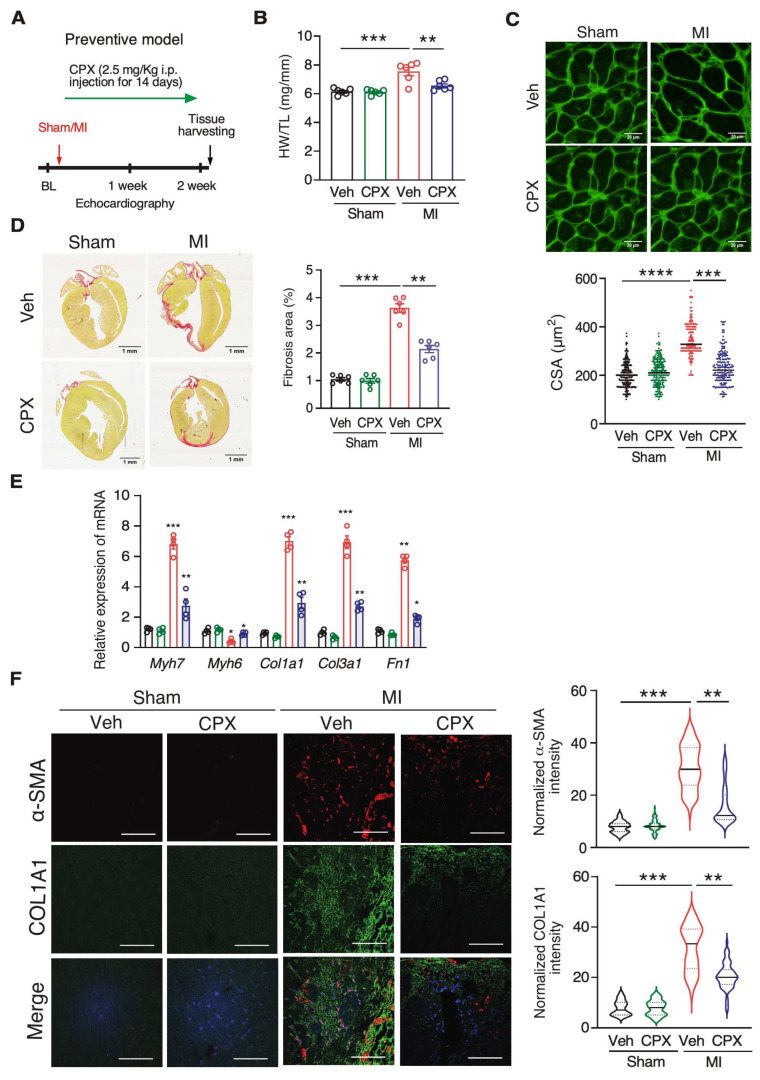
Ciclopirox antagonizes cardiac hypertrophy and fibrosis after MI in a preventive model. (**A**) Schematic of a preventive model using CPX to treat MI in mice. (**B**) Reduced HW/TL ratio in 2-week MI mouse HF model compared to vehicle-treated mice. *n* = 9/9/7/9 for the four groups of Veh Sham/CPX Sham/Veh MI/CPX MI. (**C**) Wheat germ agglutinin staining of the vehicle and CPX-treated mice with sham or MI surgery. The cross-sectional area (CSA) of CMs was measured and quantified. *n* = 6 hearts per group with 50–100 CMs measured per heart. Scale bar: 20 μM. (**D**) Picrosirius red staining indicates a decreased fibrotic area in the hearts of CPX-treated mice after MI surgery. Scale bar: 1 mm. *n* = 6 for all the groups. (**E**) RT-qPCR measurement of expression of cardiac hypertrophy and fibrosis marker mRNAs. 18S rRNA was used as a normalizer. *n* = 4 for all the groups. (**F**) α-SMA and COL1A1 protein expression in heart tissues of the vehicle and CPX-treated mice with sham or MI surgery. *n* = 5 hearts per group with >300 CFs measured per heart. Scale bar: 50 μM. * *p* < 0.05, ** *p* < 0.01, *** *p* < 0.001, **** *p* < 0.0001 by Two-way ANOVA with Tukey’s multiple comparisons test.

**Figure 3:**
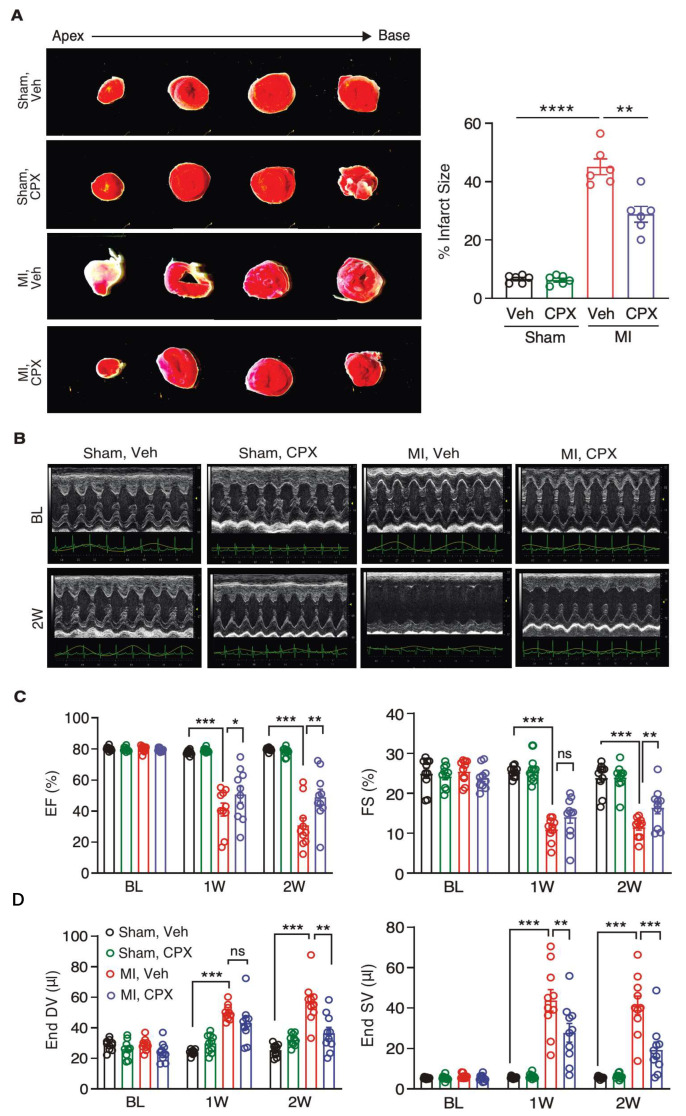
Ciclopirox reduces scar formation and preserves cardiac function after MI surgery. (**A**) TTC (2,3,5-Triphenyltetrazolium chloride) staining of hearts of vehicle and CPX-treated mice after 14 days post-Sham or MI surgery (*n* = 6 per group). The infarct area was calculated from the apex to the base of the heart sections. (**B**) Representative echocardiographic images of the vehicle and CPX-treated mice after Sham or MI surgery. (**C**) Ejection fraction (EF) and fractional shortening (FS) were partially recovered in CPX-treated mice in MI surgery-induced mouse HF model. *n* = 10/10/10/10 for the four groups of Veh Sham/CPX Sham/Veh MI/CPX MI. (**D**) End systolic and diastolic volumes were reduced in CPX-treated mice in MI surgery-induced mouse HF model. * *p* < 0.05, ** *p* < 0.01, *** *p* < 0.001, **** *p* < 0.0001 by Two-way ANOVA with Tukey’s multiple comparisons test.

eIF5A is essential for translating Pro-rich proteins [6,7,8,9], which include many extracellular matrix proteins such as collagens. To test whether inhibition of eIF5A activity reduces cardiac fibrosis, we treated the MI mouse model with a deoxyhypusine hydroxylase inhibitor CPX after the MI surgery (4 h post-MI) for 14 consecutive days (Figure 2A). We monitored the phenotypic improvement over the vehicle control treatment. We observed significantly reduced cardiac hypertrophy, as shown by the reduced ratio of heart weight to tibia length (HW/TL) and cardiomyocyte cell surface area (Figure 2B,C). The area of cardiac fibrosis was decreased by ~40% after CPX treatment (Figure 2D). Expressions of cardiac hypertrophy marker genes *Myh7* and *Myh6* were dysregulated upon MI surgery and partially restored by CPX treatment. Moreover, *Col1a1*, *Col1a3,* and *Fn1* mRNA expression levels were induced during MI and significantly reduced by CPX (Figure 2E, Table 2). Immunostaining of myofibroblast activation marker proteins α-SMA and COL1A1 further confirmed reduced cardiac fibrosis at the protein level, as both proteins were drastically reduced by 66% and 39% upon CPX treatment following MI, respectively (Figure 2F). Consistently, the infarct size was reduced by 36% after CPX treatment following MI (Figure 3A). Echocardiography measurement showed that left ventricle ejection fraction and fractional shortening were partially recovered in CPX-treated mice compared to vehicle-treated mice after MI surgery (Figure 3B,C). In addition, left ventricle end diastolic and systolic volumes were reduced in CPX-treated mice (Figure 3C). Taken together, these results suggest that CPX prevents hearts from cardiomyocyte hypertrophy and cardiac fibrosis, and restores cardiac function after MI insult.

### 3.3. Ciclopirox Treatment Reverses Cardiac Pathological Remodeling and Reduces Fibrosis

**Figure 4:**
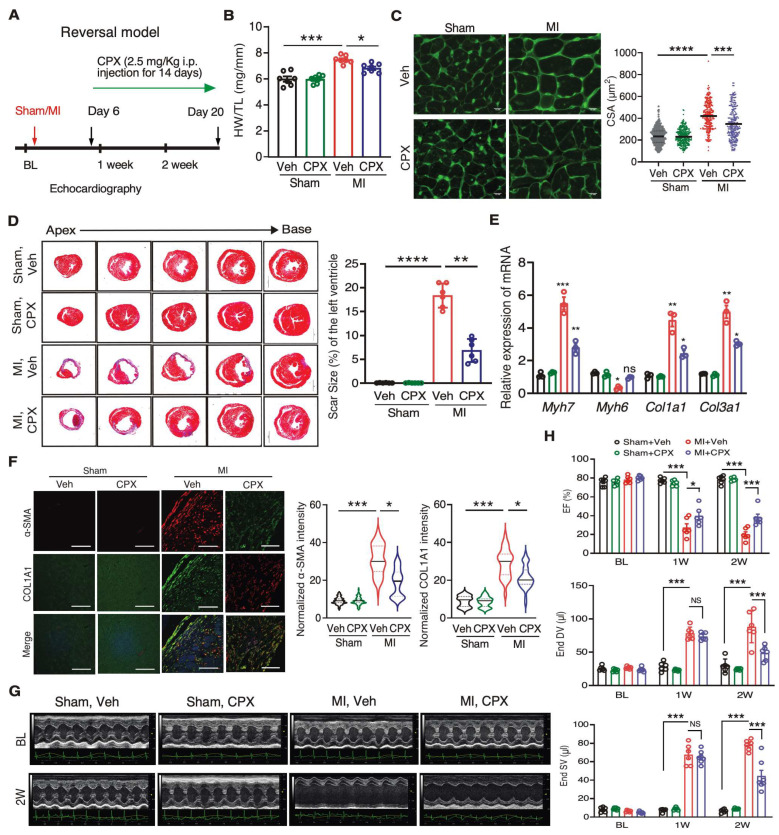
Ciclopirox reduces cardiac remodeling and fibrosis after MI in a reversal model. (**A**) Schematic of a reversal model using CPX to treat MI in mice. (**B**) Reduced HW/TL ratio in 3-week MI mouse HF model compared to vehicle-treated mice. *n* = 7 for the four groups of Veh Sham/CPX Sham/Veh MI/CPX MI. (**C**) Wheat germ agglutinin staining of the vehicle and CPX-treated mice with sham or MI surgery. The cross-sectional area (CSA) of CMs was measured and quantified. *n* = 6 hearts per group with 250–300 CMs measured per heart. Scale bar: 10 μM. (**D**) Trichrome blue staining indicates a decreased fibrotic area in the hearts of CPX-treated mice after MI surgery. Scale bar: 1 mm. *n* = 6 for all the groups. (**E**) RT-qPCR measurement of expression of cardiac hypertrophy and fibrosis marker mRNAs. 18S rRNA was used as a normalizer. *n* = 3 for all the groups. (**F**) α-SMA and COL1A1 protein expression in heart tissues of CPX-treated mice with MI surgery. *n* = 5 hearts per group with >300 CFs measured per heart. Scale bar: 50 μM. (**G**) Representative echocardiographic images of the vehicle and CPX-treated mice after sham or MI surgery. (**H**) Ejection fraction (EF) was partially recovered, and end systolic and diastolic volumes were reduced in CPX-treated mice in MI surgery-induced mouse HF model. *n* = 6 for the four groups of Veh Sham/CPX Sham/Veh MI/CPX MI. * *p* < 0.05, ** *p* < 0.01, *** *p* < 0.001, **** *p* < 0.0001, ns: not significant by Two-way ANOVA with Tukey’s multiple comparisons test.

To examine the potential therapeutic benefit of CPX in ischemic heart disease, we tested a reversal model by starting CPX injection 6 days after MI surgery for 14 consecutive doses (Figure 4A). We found that cardiac hypertrophy was reduced by CPX treatment compared to vehicle treatment following MI surgery, as indicated by decreased HW/TL ratio and cell surface area of cardiomyocytes by 17% (Figure 4B,C). Intriguingly, the fibrosis area was reduced by 62%, indicated by Mason’s Trichrome staining (Figure 4D). The expression of cardiomyocyte hypertrophy markers *Myh7* and *Myh6* was partially reversed by CPX, while the expression of fibrosis markers *Col1a1* and *Col3a1* was reduced by ~50% (Figure 4E, Table 2). Consistently, myofibroblast marker protein COL1A1 and α-SMA levels were significantly decreased in the CPX treatment group (Figure 4F). Echocardiographic analysis showed that cardiac function was moderately enhanced, as indicated by increased ejection fraction and decreased left ventricle end diastolic and systolic volume upon CPX treatment, 20 days after initial MI surgery (Figure 4G,H). These results suggest that CPX antagonizes cardiac pathological remodeling and fibrosis in MI heart failure models.

### 3.4. eIF5A Inhibition by CPX Blocks Cardiac Fibroblast-to-Myofibroblast Activation

**Figure 5:**
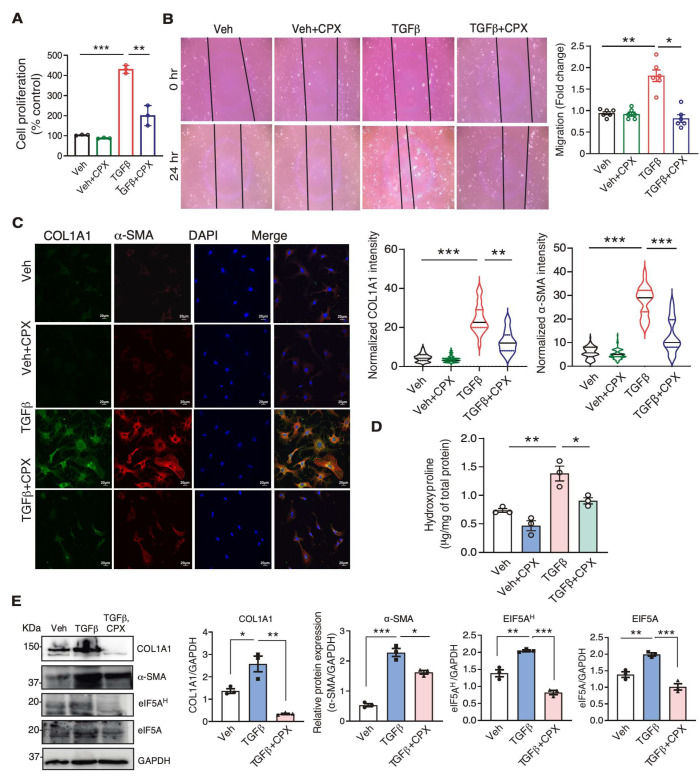
Ciclopirox inhibits cardiac fibroblast activation, migration, and ECM protein expression. (**A**) The cell proliferation rate of Veh., CPX, TGFβ, TGFβ + CPX treated primary mouse CFs. (**B**) Representative images of migrating cells of the vehicle and CPX-treated primary mouse CFs after vehicle or TGFβ treatment. Quantification of scratch closure after 24 h was shown. *n* = 6 for each group, and each experiment was repeated 3 times. (**C**) IF quantification of COL1A1 and α-SMA protein expression after CPX treatment in TGFβ-treated primary mouse CFs. *n* = 120–150 cells from three biological replicates were analyzed. Scale bar: 20 μM. Representative images were shown. (**D**) Measurement of hydroxyl-proline in the vehicle and CPX-treated primary mouse CFs after vehicle or TGFβ treatment. *n* = 3 for all the groups. (**E**) Western blot measurement of protein expression of COL1A1, α-SMA, hypusinated eIF5A, and eIF5A in the vehicle and CPX-treated primary mouse CFs after vehicle or TGFβ treatment. Quantification of protein expression level was shown. *n* = 3 for each group. * *p* < 0.05, ** *p* < 0.01, *** *p* < 0.001 by Two-way ANOVA with Tukey’s multiple comparisons test.

Because CPX inhibited cardiac fibrosis in the mouse MI model in vivo, we sought to test whether CPX directly acts on the cardiac fibroblast cells autonomously. We treated cultured primary mouse CFs with CPX following TGFβ stimulation, and examined the changes in cellular phenotypes. TGFβ induced cell proliferation by 4.2 folds, while CPX reduced the proliferation rate by 53% (Figure 5A). In a wound scratch assay, we observed that CPX significantly decreased TGFβ-induced cell migration of primary mouse CFs to the basal level (Figure 5B). In the cultured mouse CF cells, COL1A1 and α-SMA protein expression were drastically reduced by ~45% and ~60%, respectively, in CPX-treated cells with TGFβ stimulation compared to TGFβ-treated cells (Figure 5C, Table 1). The global cellular incorporation of hydroxyproline was reduced by 35%, indicating remarkably reduced total collagen synthesis (Figure 5D). Using Western blotting, we observed that expression of eIF5A^H^, COL1A1, and α-SMA was indeed significantly reduced in primary mouse CFs treated with CPX following TGFβ stimulation (Figure 5E, Table 1). Taken together, these data suggest that CPX directly inhibits the cellular activity of cardiac fibroblasts, prevents fibroblast-to-myofibroblast transition, and compromises the synthesis of collagen proteins.

### 3.5. CPX Reduces the Expression of Pro-Rich Proteins and Alters Proteome in Cardiac Fibroblasts

**Figure 6:**
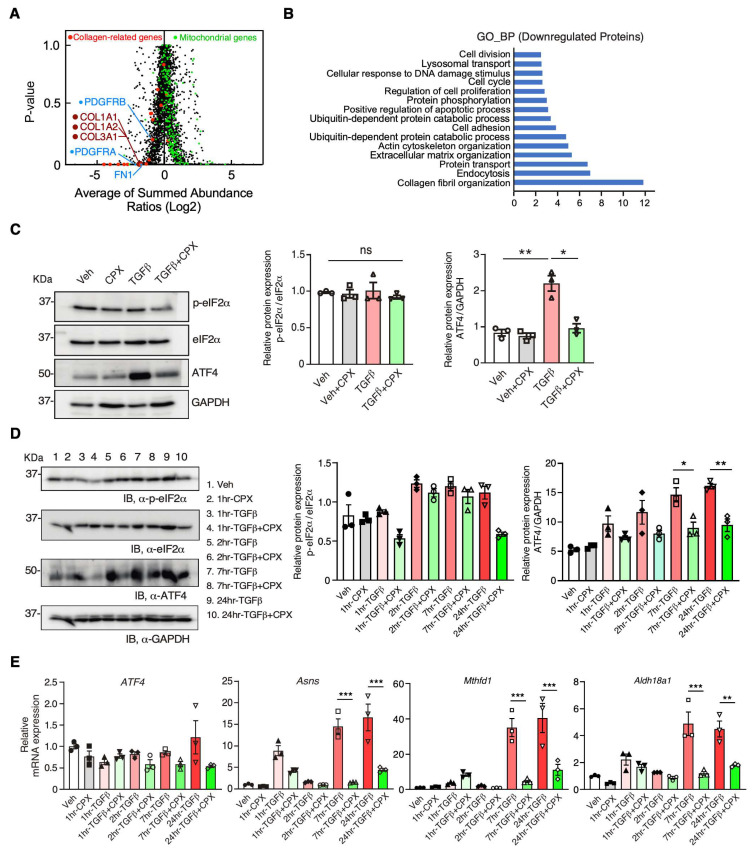
Proteomic reprogramming by ciclopirox in mouse cardiac fibroblasts. (**A**) Mass spectrometry analysis in 10 μM CPX-treated primary mouse CFs compared to vehicle-treated cells (24 h treatment). (**B**) Gene ontology analysis of downregulated proteins in primary mouse CFs by CPX treatment. (**C**) Western blot analysis of p-eIF2α, eIF2α, and ATF4 protein expression in NIH/3T3 cells. NIH/3T3 cells were treated with TGFβ (10 nM) concentration and CPX (10 μM) at 24 h. Quantification of WB was shown on the right panel. (**D**) Western blot analysis of p-eIF2α, eIF2α, and ATF4 protein expression in NIH/3T3 cells. NIH/3T3 cells were treated with TGFβ (10 nM) concentration and CPX (10 μM) at 1, 2, 7, and 24 h in a time course. Quantification of WB was shown on the right panel. (**E**) RT-qPCR quantification of *ATF4* mRNA and ATF4 target gene expression. *n* = 3 of independent experiments. * *p* < 0.05, ** *p* < 0.01, *** *p* < 0.001, ns: not significant by Two-way ANOVA with Tukey’s multiple comparisons test.

eIF5A is known to promote Pro-rich protein synthesis [7,8] and may affect extracellular matrix protein expression, among others [3]. To investigate the effect of CPX on global proteomic reprogramming, we performed quantitative mass spectrometry analysis in primary mouse CFs under treatment of CPX or vehicle at baseline (Figure 6A, Supplementary Table S1). Based on gene ontology (GO) analysis, we found that in CF cells, several cellular pathways enriched from 520 downregulated proteins (Log_2_FC < −0.5) were suppressed, including multiple collagen-related proteins being significantly reduced in CPX-treated CFs, such as three major fibrillar collagens COL1A1, COL1A2, COL3A1, profibrotic receptors PDGFRA and PDGFRB, and fibronectin (FN1) (Figure 6A, Supplementary Table S1). Moreover, we identified 317 out of 520 downregulated proteins (Log_2_FC < −0.5) by CPX treatment containing Pro-rich codons after overlapping with our established database of Pro-Pro motif-containing genes (Supplementary Table S1) [3], suggesting CPX inhibition of eIF5A activity reduces Pro-rich protein expression. The top GO pathways enriched from these 317 Pro-rich codon-containing proteins are collagen fibril organization (including COL14A1, COL1A1, COL1A2, COL3A1, COL2A1, COL5A1, COL5A2, COL11A1, P3H4, LOXL1, LOXL2, LOX, NF1, CYP1B1), endocytosis, protein transport, actin cytoskeleton organization, cell adhesion, cell migration, cell proliferation, among others (Supplementary Table S1). In contrast, the major GO pathways enriched from the 760 upregulated proteins (Log_2_FC > 0.5) include RNA splicing, mitochondrial translation, ribosome biogenesis, and mRNA transport. Intriguingly, many nuclear-encoded mitochondrial proteins were upregulated (Figure 6A, highlighted in green; Supplementary Table S1), including pathways of lipid metabolic process, mitochondria organization, tricarboxylic acid cycle, ATP metabolic process, glutathione metabolic process, and amino acid metabolic pathways (Supplementary Table S1). These findings indicate a potential compensatory effect to maintain the viability of CFs when Pro-rich protein expression (e.g., ECM) is suppressed.

We hypothesize that CPX-mediated inhibition of Pro-rich mRNA translation may trigger ribosome pausing or stalling [6]. Ribosome stalling due to arrested translation elongation is known to activate the integrated stress response (ISR), which also inhibits cap-dependent mRNA translation and activates *ATF4* mRNA translation [21]. Therefore, we examined the markers of ISR, including phosphorylation of eukaryotic initiation factor 2α (eIF2α), and activation of ATF4 expression. We did not observe any increase in phosphor-eIF2α or eIF2α protein expression (Figure 6C,D, Table 1), suggesting no strong ribosome stalling and subsequent ISR activation. Instead, ATF4 protein expression was robustly induced by TGFβ stimulation and blocked by CPX treatment (Figure 6C,D, Table 1). Interestingly, a bioinformatic search revealed that ATF4 contains multiple Pro-rich codons in an evolutionarily conserved region (i.e., P^241^PDNLPSPGGSRGSPRPKPYDPPG^264^ in mice and P^242^NRSLPSPGVLCGSARPKPYDPPG^265^ in humans) [3], and could be sensitive to CPX inhibition of eIF5A activity. Consistently, the expression of multiple classic ATF4 target genes was consistently increased under TGFβ treatment and decreased by CPX, including *Asns*, *Mthfd1*, and *Aldh18a1* (Figure 6E, Table 2). These results indicate that CPX can reprogram the cellular proteome and influence the cellular function of CFs by inhibiting the expression of Pro-rich proteins such as collagens and ATF4, among other proteins.

## 4. Discussion

In this study, we discovered that eIF5A hypusination is induced in failing human and mouse hearts undergoing pathological cardiac remodeling. As an eIF5A hypusination inhibitor, CPX reduces cardiac hypertrophy and fibrosis and improves cardiac functions in mouse preventive and reversal MI models. CPX effectively inhibits primary mouse CF proliferation, migration, and CF-to-myofibroblast trans-differentiation. Moreover, mass spectrometry-based proteomic analysis shows that ECM proteins are enriched in the downregulated proteins by CPX treatment in mouse CFs. Mechanistically, CPX inhibits eIF5A activation and reduces translation elongation of Pro-rich proteins, including ECM protein and a metabolic regulatory transcription factor ATF4. Thus, reprogramming proteome and metabolic gene expression protects the heart from ischemic cardiac injury.

eIF5A is an essential housekeeping enzyme for translation elongation during ribosome-mediated protein synthesis [6,9]. eIF5A catalyzes peptide bond formation between two consecutive proline amino acids for the efficient synthesis of ProPro-containing proteins. eIF5A hypusination on a Lys^50^ residue is essential for its catalytic function. eIF5A hypusination forms through two-step enzymatic reactions that are mediated by deoxyhypusine synthase and deoxyhypusine hydroxylase (DOHH) [11]. DOHH enzyme requires iron to catalyze the formation of hypusine modification of the lysine residue in eIF5A [13]. CPX is well known to chelate iron in eukaryotic cells [22]. CPX treatment inhibits DOHH enzymatic activity and reduces eIF5A hypusination and activity, thereby reducing Pro-rich protein expression. Based on our mass spectrometry screening, we discovered that Pro-rich proteins are reduced in their expression levels by CPX (Figure 6A,B). ATF4 protein contains multiple Pro-rich motifs, and thus, it is also sensitive to CPX treatment (Figure 6C,D). Our studies suggest that CPX inhibition of eIF5A is beneficial in treating cardiac pathological remodeling. However, the mechanism of how eIF5A hypusination is increased in MI or heart failure remains unclear. Spermidine is a well-known metabolite precursor for hypusination [23]. We speculate that spermidine level [24] or DOHH enzyme expression level may be induced by MI stress, leading to an increased level of eFI5A^H^. The regulatory mechanism of eIF5A^H^ modification warrants future studies.

CPX was used in phase I clinical trials in patients with advanced hematologic malignancies daily. This trial showed a promising outcome of either hematologic improvement or disease stabilization in ~67% of patients without having apparent toxicity [25]. CPX is also used for intravenous administration to treat advanced solid tumors in clinical trial phase I (ClinicalTrial.gov; Safety, dose tolerance, pharmacokinetics, and pharmacodynamics study of CPX-POM in patients with advanced solid tumors). CPX has been shown to enhance the response to inotropic stimulation in aged cardiomyocytes through upregulation of hypoxia-inducible factor 1 (HIF-1) in rat cardiomyocytes [16]. Additionally, CPX-induced HIF-1α increases the expression of urocortin 2, which elevates myocardial contractility and prevents myocyte apoptosis under ischemic stress [26]. Here, we show the cardioprotective effect of CPX against cardiac fibroblast activation in vitro and in vivo. Our study used CPX to treat a mouse MI heart failure model for the first time, and showed the promise of using CPX to treat cardiac disease in vivo. This work suggests that CPX can be repurposed to treat cardiac fibrosis and ischemic heart disease.

Due to the iron (Fe^3+^) chelating activity of CPX, it has multiple downstream effects, e.g., inhibiting iron-dependent enzymes such as DOHH, PHD (proline 4-hydroxylase for hypoxia-inducible factor-1α activation), RR (ribonucleotide reductase), and CDKs (cyclin-dependent kinases). Therefore, we cannot rule out the dependence of the anti-fibrotic effects of CPX on other pathways. However, we observed ~61% of ProPro peptide-bearing proteins out of 520 downregulated proteins at the steady state levels detected by mass spectrometry proteomic analysis. GO analysis results demonstrate that Pro-rich ECM proteins are mostly highly enriched as downregulated proteins, which align well with the outcome of CF-to-myofibroblast activation assays after CPX treatment (Figure 5C–E and Figure 6A,B). Moreover, ProPro-containing proteins are reduced in cell proliferation and migration pathways, consistent with in vitro primary mouse CF cell culture phenotyping data (Figure 5A,B and Figure 6B). As a compensatory response, upregulated pathways lie mainly in mRNA metabolism and translation, and mitochondrial metabolic and energetic pathways that may prevent CF cell apoptosis and death, thereby minimizing inflammatory responses. Altogether, these findings confirm that CPX-driven reduced eIF5A hypusination and activity are major contributors to anti-fibrotic effects, among other potential pathways.

## 5. Novelty and Significance

What is known?

- eIF5A is an essential housekeeping enzyme required for translation elongation during ribosome-mediated protein synthesis, especially peptide bond formation between two consecutive proline amino acids for efficient synthesis of ProPro-containing proteins;
- eIF5A hypusination on a Lys^50^ residue is essential for its catalytic function;
- eIF5A hypusination forms during a two-step enzymatic reaction that is mediated by deoxyhypusine synthase and deoxyhypusine hydroxylase (DOHH);
- eIF5A plays a vital role in inflammation, such as macrophage activation and cancer progression;
- Ciclopirox, a deoxyhypusine hydroxylase inhibitor, is an FDA-approved drug to treat fungal infections.
- What New Information Does This Article Contribute?
- eIF5A hypusination is induced in failing human and mouse hearts, and mouse hearts undergoing pathological cardiac remodeling;
- Ciclopirox is repurposed to treat cardiac fibrosis in the mouse myocardial infarction model;
- Ciclopirox reduces cardiac hypertrophy and fibrosis, and partially preserves cardiac functions in the mouse myocardial infarction model;
- Mass spectrometry-based proteomic analysis shows that extracellular matrix proteins are significantly enriched in the downregulated proteins by ciclopirox treatment in mouse cardiac fibroblast cells;
- Critical metabolism regulatory transcription factor ATF4 is significantly downregulated by ciclopirox in activated CFs as a Pro-rich protein.

## Figures and Tables

**Table 1:** Antibodies used in this study.

Primary Antibody	Cat. No. and Vendor	Dilution	Detection
Mouse anti-GAPDH	60004-1-lg; ProteinTech	WB (1:5000)	Multiple cell types
Mouse anti-eIF5A	SC-390062; Santa Cruz Biotechnology	WB (1:500)	Mouse and human CFs and heart tissues and sections
Rabbit anti-eIF5A^H^	ABS1064-1; EMD Millipore	WB (1:1000)	Mouse and human CFs and heart tissues and sections
Goat anti-Mouse AF488	A11001; Life Technologies	IF (1:500)	Multiple cell types
Goat anti-Rabbit AF488	A11034; Life Technologies	IF (1:500)	Multiple cell types
Goat anti-Mouse AF594	A1105; Invitrogen	IF (1:500)	Multiple cell types
Goat anti-Rabbit AF594	A11012; Invitrogen	IF (1:500)	Multiple cell types
Rabbit anti-COL1A1	SAB2109131; Sigma Aldrich	IF (1:300)	Myofibroblasts (MFs)
Mouse anti-Periostin	66491-1-lg; ProteinTech	IF (1:500)	Mouse and human heart tissue sections; MFs
Mouse anti-a-SMA (monoclonal)	A2547; Sigma Aldrich	IF (1:300) WB (1:1000)	Mouse and human heart tissue sections; MFs
Goat anti-a-SMA (polyclonal)	PA5-18292; ThermoFisher	IF (1:500)	Mouse and human heart tissue sections; MFs
Rabbit anti-p-eIF2a S51	3597S; Cell Signaling Technology	WB (1:1000)	NIH/3T3 mouse fibroblast cells
Mouse anti-eIF2a	2103S; Cell Signaling Technology	WB (1:1000)	NIH/3T3 mouse fibroblast cells
ATF4	11815S; Cell Signaling Technology	WB (1:1000)	NIH/3T3 mouse fibroblast cells
Wheat Germ Agglutinin AF-488	W11261; Invitrogen	IF (1:1000)	Mouse heart tissue sections

**Table 2:** qPCR primers used in this study (SYBR green).

SYBR Green RT-qPCR Primers			
Species	Target	Forward Primer (5′–3′)	Reverse Primer (5′–3′)
Mouse	Fn1	TCCTGTCTACCTCACAGACTAC	GTCTACTCCACCGAACAACAA
Mouse	Myh6	GGAGGAGTATGTTAAGGCCAAG	CATCACCTGGTCCTCCTTTATG
Mouse	Myh7	CCACCCAAGTTCGACAAGAT	AAGAGGCCCGAGTAGGTATAG
Mouse	Gapdh	AACAGCAACTCCCACTCTTC	CCTGTTGCTGTAGCCGTATT
Mouse	Col1a1	CGCCATCAAGGTCTACTGCAA	CTCGCTTCCGTACTCGAAC
Mouse	Col3a1	TAAAATTCTGCCACCCCGAAC	TGCACCAGAATCTGTCCAC
Mouse	RN18S	TGACTCAACACGGGAAACCTC	CATGCCAGAGTCTCGTTCGTT
Mouse	Atf4	GGCCAAGCACTTGAAACCTC	AACATCCAATCTGTCCCGGAA
Mouse	Asns	GCAGTGTCTGAGTGCGATGAA	TCTTATCGGCTGCATTCCAAAC
Mouse	Mthfd1	CTGCTGCTAACAACCTCGT	AGTCTTCTCAATGCCTAGCC
Mouse	Aldh18a1	AATCAGGGCCGAGAGATGATG	GGCCTCTAAGACCGGAATTGC

## Data Availability

Data is contained within the article or Supplementary Material. The data presented in this study are available in this article and related Supplementary Material.

## Supplementary Materials

The following supporting information can be downloaded at: https://www.mdpi.com/article/10.3390/jcdd10020052/s1, Table S1: Mass spectrometry analysis of CPX- versus vehicle treated primary mouse cardiac fibroblasts.
